# Polymorphisms of the murine mitochondrial *ND4*, *CYTB* and *COX3* genes impact hematopoiesis during aging

**DOI:** 10.18632/oncotarget.11952

**Published:** 2016-09-10

**Authors:** Christin Kretzschmar, Catrin Roolf, Katrin Timmer, Anett Sekora, Gudrun Knübel, Hugo Murua Escobar, Georg Fuellen, Saleh M. Ibrahim, Markus Tiedge, Simone Baltrusch, Robert Jaster, Rüdiger Köhling, Christian Junghanss

**Affiliations:** ^1^ Department of Medicine III - Hematology/Oncology/Palliative Care, Rostock University Medical Center, Rostock, Germany; ^2^ Institute for Biostatistics and Informatics in Medicine and Ageing Research, Rostock University Medical Center, Rostock, Germany; ^3^ Institute of Experimental Dermatology, University of Lübeck, Lübeck, Germany; ^4^ Institute of Medical Biochemistry and Molecular Biology, Rostock University Medical Center, Rostock, Germany; ^5^ Department of Medicine II, Division of Gastroenterology, Rostock University Medical Center, Rostock, Germany; ^6^ Oscar Langendorff Institute of Physiology, Rostock University Medical Center, Rostock, Germany

**Keywords:** mtDNA, aging, hematopoiesis, ROS, stem cell, Gerotarget

## Abstract

During aging, mitochondrial DNA (mtDNA) can accumulate mutations leading to increasing levels of reactive oxygen species (ROS). Increased ROS were described to activate formerly quiescent hematopoietic stem cells (HSC). Mutations in mtDNA were shown to enhance the risk for myelodysplastic syndrome and leukemia. However, the complex relationship between mtDNA variations, ROS and aging of the hematopoietic system is not fully understood.

Herein, three mouse strains with mtDNA polymorphisms in genes of respiratory chain complexes I (ND4), III (CYTB) and IV (COX3) were compared to a reference strain during aging. Analysis focused on ROS and ATP levels, bone marrow composition and blood counts. Additionally, hematopoietic restoration capacity following cytotoxic stress was tested.

Mice with polymorphisms in ND4 and CYTB gene had significantly decreasing ROS levels in bone marrow cells during aging, without effecting ATP levels. In addition, the frequency of stem and progenitor cells increased during aging but the amount of lymphocytes in the peripheral blood decreased during aging.

In summary, the presence of mtDNA polymorphisms affecting the respiratory chain complexes I, III and IV was associated with altered ROS levels as well as changes in BM and peripheral blood composition during aging.

## INTRODUCTION

Elderly individuals are frequently affected by age-related symptoms and diseases such as higher susceptibility towards infections, higher incidence of myelodysplastic syndrome (MDS) and leukemia [1]. Microenvironment and intrinsic factors were described to cause changes in hematopoietic stem cells (HSC) during aging, resulting in lineage skewing towards enhanced myelopoiesis, but impaired erythropoiesis and lymphopoiesis [2].

The major cellular source of ROS is the mitochondrial respiratory chain [3]. Elevated concentrations of ROS may damage various cell components [4–6]. Especially elevated concentrations of hydrogen peroxide (H_2_O_2_) were shown to play a crucial role as signaling molecules in different cellular processes. Antioxidants like thioredoxines or peroxiredoxins were suggested not only to serve as ROS scavengers but to function as sensors and effectors influencing various processes as apoptosis and cell growth [7].

Mitochondrial DNA (mtDNA) accumulates mutations during aging possibly causing concomitant mitochondrial dysfunction [8,9]. Mutations in mtDNA have been linked to impaired hematopoiesis and the progression of malignancies. Mitochondrial genome instability was correlated with increased intestinal tumorigenesis in a cancer developing mouse model [10]. Also, distinct mtDNA mutations were shown to enhance levels of reactive oxygen species (ROS) and tumorigenicity in prostate cancer [11] as well as metastasis in lung cancer cells [12]. Moreover, a pathogenic deletion in mtDNA led to decreased repopulating capacity of murine HSCs in a competitive transplantation model [13]. Further, the ablation of proof reading of DNA polymerase gamma was shown to give rise to extended mtDNA mutations followed by premature aging effects on hematopoiesis [14]. Additionally, elevated levels of ROS promoted progress from MDS to acute leukemia in a transgenic MDS mouse model [15].

Overall, the complex relationship between mtDNA variations, ROS and the aging hematopoietic system is not fully understood.

Conplastic mice strains are a well suitable model system for the investigation of specific mtDNA variations and their influence on ROS and ATP levels, as well as the investigation of tissues and aging of the whole organism [16]. Here, we investigated novel conplastic mouse strains C57BL/6Ntac-mt^AKR/J^ (mtAKR), C57BL/6Ntac-mt^129S1SvlmJ^ (mt129S1) and C57BL/6Ntac-mt^NOD/LTJ^ (mtNOD) as well as the background strain C57BL/6Ntac (B6Ntac) in regards to their hematopoietic changes during aging. All mice harbor different polymorphisms in the genes of the respiratory chain. The B6Ntac mice harbored a polymorphism in the ND4 gene, encoding for a subunit of respiratory chain complex I. The mt129S1 strain differed from mtAKR in the CYTB gene affecting complex III, while mtNOD strain displayed a polymorphism in COX3 gene (complex IV) and in the tRNA-Arg. The mtAKR strain served as reference for the mtDNA (Table 1).

We hypothesized that the presence of polymorphisms in the respiratory chain genes influences aging of the hematopoietic system. Young (3 months), mid-aged (12 months) and advanced-aged mice (24 months) were analyzed with focus on basal ROS and ATP levels in bone marrow (BM). Further, hematopoietic subpopulations were characterized in the BM as well in the peripheral blood. Moreover, the recovering potential of hematopoietic stem cells following cytostatic stress was investigated. Our results revealed that the presence of mtDNA polymorphisms in the ND4, CYTB and COX3 genes are associated with decreasing intracellular ROS levels as well as decreasing lymphocyte counts during aging.

## RESULTS

### ROS and ATP levels

**Figure 1:**
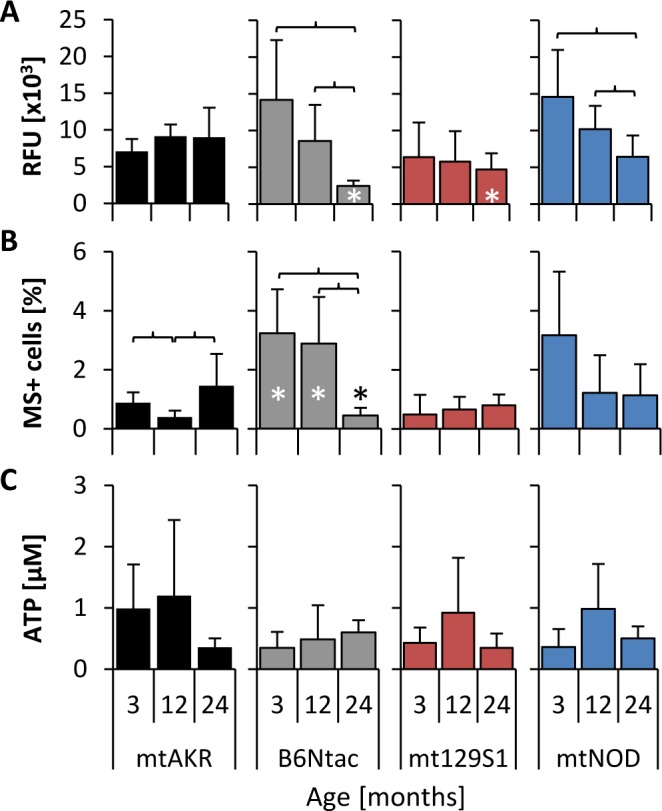
Polymorphisms in mtDNA altered basal levels of ROS Levels of complete intracellular ROS **A.**, mitochondrial superoxide **B.** and ATP **C.** were analyzed in isolated bone marrow at three aging stages to investigate changes during aging. The asterisk (*) marks significant differences (at a *p*-value threshold of <0.05) *versus* the mtAKR strain at the same aging stage. Brackets indicate significant changes within one strain during aging. Six or more mice were investigated for each strain and aging stage. Detailed numbers of animals are summarized in supplement tables 1-3.

ROS levels in BM cells were investigated by two different methods. While DCFH-DA is used to detect the total intracellular ROS (Figure 1A), MitoSOX™ (MS) specifically detects the mitochondrial superoxide (Figure 1B).

**Table 1:** Investigated mouse strains with polymorphisms in mtDNA

Strain	Variation in mtDNA	Affected Gene	Aminoacid Exchange	Reference mtDNA (Pubmed GenBank)
mtAKR	reference	−	−	EF108332.1
B6Ntac	nt 11516 A/G	ND4	Asn-Ser	JF286601.1
mt129S1	nt 15124 A/G	CYTB	Ile-Val	EF108330.1
mtNOD	nt 9348 G/A nt 9821 9A/10A	COX3 tRNA-Arg	Val-Ile -	EF108340.1

From 3 to 24 months, the reference strain mtAKR revealed constant levels of intracellular ROS (7.1 ± 1.7 x10^3^ to 9.0 ± 4.0 x10^3^ relative fluorescence units - RFU) and mitochondrial superoxide (0.9 ± 0.4 % to 1.5 ± 1.1 % of total nucleated cells -TNC). The B6Ntac strain displayed higher levels of total ROS and mitochondrial superoxide at 3 months (14.2 ± 8.1 x10^3^ RFU; 3.2 ± 1.5 %) compared to 24 month old animals. However, at 24 months both parameters showed significantly lower levels than the reference strain (2.5 ± 0.7 x10^3^ RFU; 0.5 ± 0.3 %). During aging ROS and mitochondrial superoxide production significantly decreased in B6Ntac. The mt129S1 strain exhibited a constant total ROS production during aging (6.4 ± 4.7 x10^3^ to 4.7 ± 2.2 x10^3^ RFU). Levels of total ROS (14.6 ± 6.4 x10^3^ to 6.4 ± 2.9 x10^3^ RFU) in BM cells of mtNOD mice decreased significantly during aging. ATP levels in all three strains did not change significantly during aging (Figure 1C).

### Subpopulations in bone marrow

**Figure 2:**
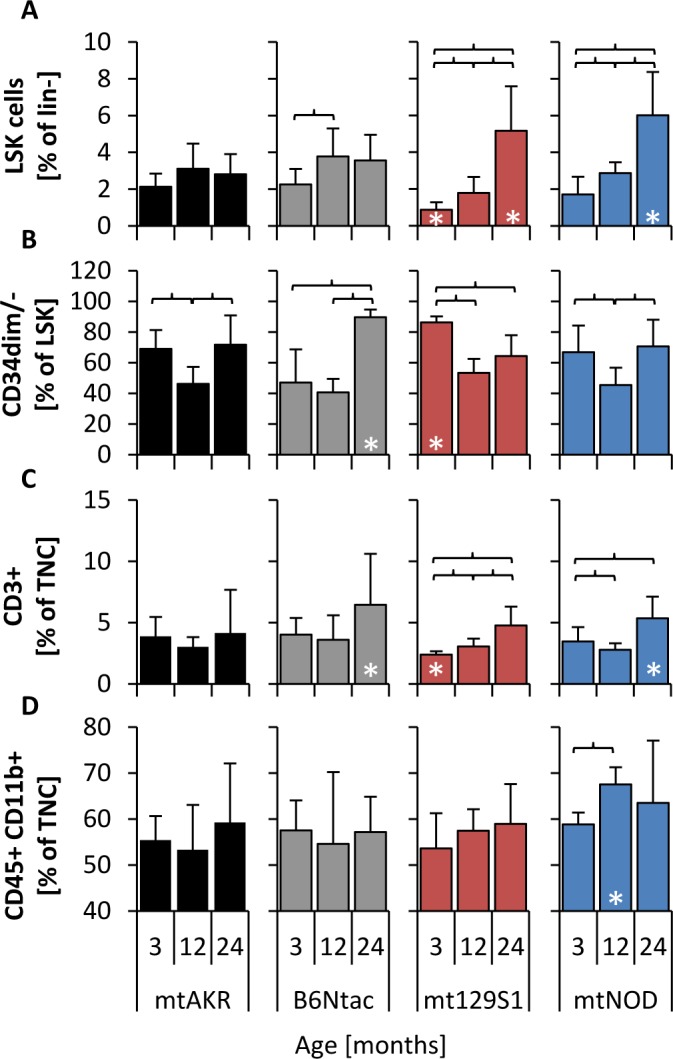
Polymorphisms in mtDNA impacted size of LSK pool and proportion of CD34^dim/−^ cells Analysis of subpopulations was carried out at three aging stages (3, 12 and 24 months) to investigate changes of bone marrow constitution during aging. LSK cells were analyzed by gating all cells negative for lineage specific antigens (lin^−^) and characterizing cells positive for Sca-1 and c-kit (Supplement 1) **A.** Long-term HSCs were investigated by determining LSK cells that are negative or weakly positive for CD34 **B.** T cells **C.** were characterized by antigen CD3 in TNC pool (total nucleated cells), while monocytes **D.** were identified by antigens CD45 and CD11b. The asterisk (*) marks significant differences (at a *p*-value threshold of <0.05) *versus* the mtAKR strain at the same aging stage. Brackets indicate significant changes within one strain during aging. Six or more mice were investigated for each strain and aging stage.

Subpopulations of BM cells were characterized by immunophenotyping during aging (Figure 2). The reference strain mtAKR displayed no significant changes of LSK (lineage^−^, Sca-1^+^, c-kit^+^) cell amount from 3 to 24 months (2.1 ± 0.7 % to 2.8 ± 1.1 % of lineage^−^ cells), neither differed the proportion of CD34^dim/−^ cells in the LSK population (69.0 ± 12.3 % to 71.8 ± 19.2 % of LSK) and the amount of T cells (3.9 ± 1.6 % to 4.2 ± 3.5 % of TNC) significantly during aging. The B6Ntac mice showed similar amounts of LSK cells during aging (2.3 ± 0.8 to 3.6 ± 1.4 %) as mtAKR, while the proportion of CD34^dim/−^ cells and the amount of T cells increased during aging to significant higher levels at 24 months (CD34^dim/−^: 89.7 ± 5.0 % of LSK; T cells: 6.5 ± 4.2 % of TNC).

Young mice of the mt129S1 strain revealed a lower amount of LSK cells (0.9 ± 0.4 % of lineage^−^ cells) with a significantly higher proportion of CD34^dim/−^ cells (86.3 ± 3.9 % of LSK) and, furthermore, a significantly lower amount of T cells (2.4 ± 0.3 % of TNC) compared to mtAKR. During aging the amount of LSK cells increased significantly in BM cells of mt129S1 strain to a significantly higher level (5.2 ± 2.4 % of lineage^−^ cells). Similarly, the mtNOD strain showed a significantly higher amount of LSK cells in aged animals (6.0 ± 2.4 % of lineage^−^ cells) compared to mtAKR. Moreover, T cells in 24 month-old mtNOD mice (5.4 ± 1.8 % of TNC) were significantly enhanced in comparison to mtAKR animals. Additionally, 12 month-old mtNOD mice displayed a significantly higher amount of monocytes in BM than mtAKR animals (67.5 ± 4.3 % vs. 53.3 ± 9.5 % of TNC). B cells and erythroid cells exhibited no considerable overall changes from 3 to 24 months (data not shown).

### Blood counts

**Figure 3:**
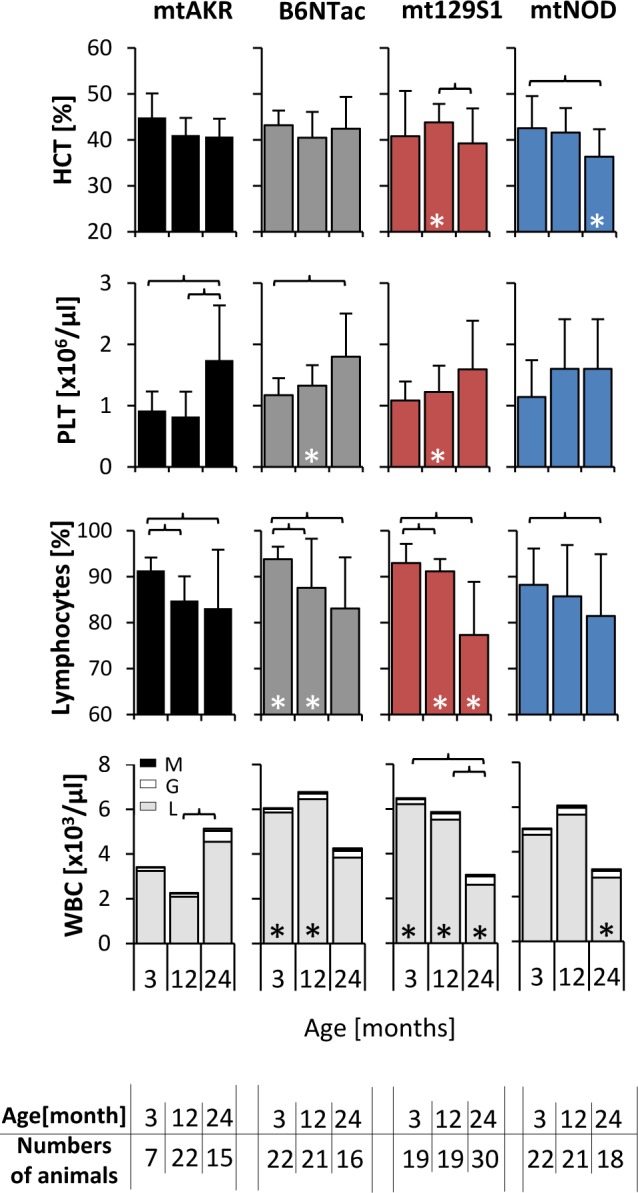
Polymorphism in mtDNA led to a reduced WBC in advanced-aged mice Whole blood was investigated at three aging stages. The asterisk (*) marks significant differences (at a *p*-value threshold of <0.05) *versus* the mtAKR strain at the same aging stage. Brackets indicate significant changes within one strain during aging. Numbers of animals investigated at each time point are displayed at the bottom. Abbreviations: M = monocytes, L = lymphocytes, G = granulocytes.

Peripheral blood counts during aging are displayed in Figure 3. The hematocrit (HCT) decreased in all four mouse strains during aging independent of the mtDNA polymorphism. Notably, the HCT of 24 month-old mtNOD mice decreased to a significantly lower level compared to mtAKR (36.3 ± 6.0 % vs. 40.7 ± 3.9 %). From 3 to 24 months, the strains displayed a uniform increase in the amount of thrombocytes (PLT) albeit not significant in all strains.

Differences in white blood cell count (WBC) i.e. total leukocytes and in particular the lymphocytes compartment were most noticeable during aging. The mtAKR mice showed a modest increase in leukocytes (WBC) from 3 to 24 months (3.5 ± 2.2 x10^3^/μl to 6.2 ± 3.3 x10^3^/μl; p = not significant). In contrast, WBC in the other three strains decreased over time, (at 24 months B6Ntac: 3.8 ± 2.1 x10^3^/μl; mt129S1: 3.3 ± 1.7 x10^3^/μl; mtNOD: 3.4 ± 2.2 x10^3^/μl). Here, WBC reduction was due to a significant decline of lymphocytes (B6NTac: 3.8 ± 3.1 x10^3^/μl; mt129S1: 2.6 ± 1.5 x10^3^/μl; mtNOD: 2.9 ± 2.1 x10^3^/μl) in the peripheral blood in 24 month old mice. Of note, absolute WBC in young mice was higher in B6Ntac and mtNOD compared to mtAKR. The total amount of neutrophils or monocytes was not significantly affected.

In summary, the polymorphisms in ND4, CYTB and COX3 gene led to a decrease of leukocytes during aging due to a reduction of lymphocytes.

**Table 2:** Nadirs of blood count parameters after 5-Fluorouracil treatment

Nadirs		mtAKR		B6Ntac		mt129S1		mtNOD	
		d	fold	d	fold	d	fold	d	fold
WBC	3m	9	0.3 ± 0.1	6	0.4 ± 0.1	9	0.4 ± 0.1	6	0.4 ± 0.2
	12m	6	0.3 ± 0.2	9	0.3 ± 0.1	6	0.3 ± 0.1	9	0.2 ± 0.2
Neutro	3m	6	0.1 ± 0.1	6	0.1 ± 0.2	6	0.1 ± 0.2	6	0.1 ± 0.2
	12m	6	0.1 ± 0.1	6	0.1 ± 0.1	6	**0.2 ± 0.1**	6	0.1 ± 0.1
Lymph	3m	9	0.3 ± 0.1	6	0.4 ± 0.1	9	0.4 ± 0.1	6	0.4 ± 0.2
	12m	6	0.3 ± 0.2	9	0.3 ± 0.2	6	0.3 ± 0.1	9	0.3 ± 0.2
Mono	3m	6	0.0 ± 0.1	6	0.0 ± 0.1	9	0.0 ± 0.0	9	0.1 ± 0.3
	12m	6	0.0 ± 0.0	9	0.0 ± 0.0	6	0.0 ± 0.1	6	0.0 ± 0.0
RBC	3m	12	0.4 ± 0.1	9	0.4 ± 0.2	12	0.5 ± 0.1	12	0.5 ± 0.2
	12m	12	0.6 ± 0.1	12	0.5 ± 0.1	12	0.6 ± 0.1	12	0.5 ± 0.2

Values of blood parameters are given as ratio to corresponding control group with standard deviation. ‘d’ indicates the first day of minimal parameter value after 5-FU administration. Bold values indicate a significant difference to mtAKR strain at same aging stage and day after 5-FU administration. Each group consisted of 10 animals.

### *In vivo* study

**Figure 4:**
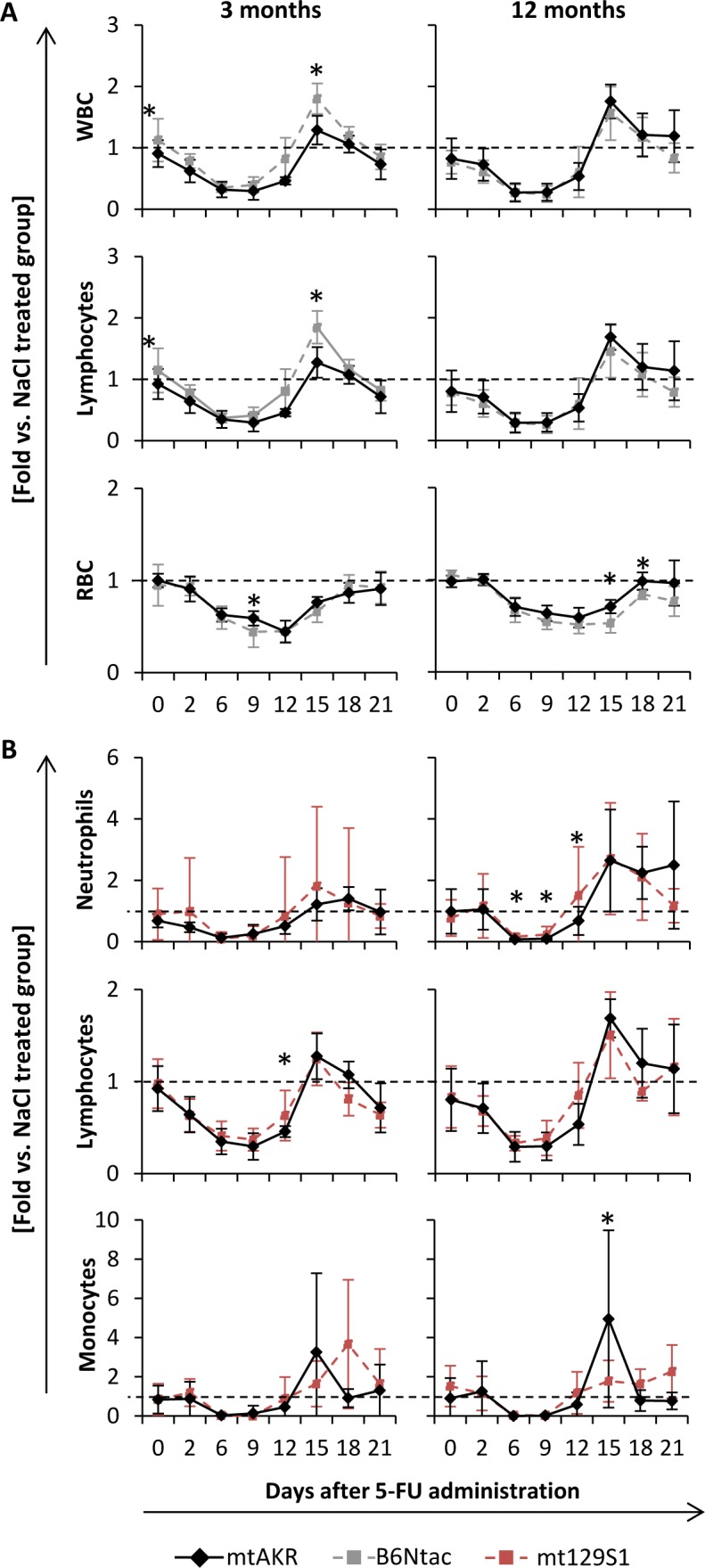
Polymorphisms in *ND4* (A) enhanced recovery of lymphocytes after cytostatic stress in young mice, while *CYTB* alteration (B) postponed recovery of monocytes Animals were treated with 5-Fluorouracil to evaluate hematopoietic restoration capacity after cytostatic stress. Blood count was measured every two to four days up to day 15 and 21, respectively. Values for leukocytes (WBC), absolute count of neutrophils, lymphocytes and monocytes as well as erythrocytes (RBC) were analyzed. Results are given as ratio to the corresponding control group treated with saline (same strain and age). Each group consisted of 10 animals. Of these, half was sacrificed at day 15 and the other half at day 21 for further investigations of bone marrow. Unexpected death occurred in one mt129S1 mouse (d1) leading to respective fewer data points. Significant differences to mtAKR strain were indicated by an asterisk (*) at a *p*-value threshold of <0.05.

Treatment with 5-Fluorouracile (5-FU) led to a pancytopenia in all four strains. However, the B6Ntac and mt129S1 strain revealed some differences during regeneration phase compared to mtAKR (Figure 4A). Ratio of WBC of 5-FU treated animals with corresponding control group decreased in all four strains and reached the nadir between day 6 and 9 with similar values (Table 2). The restoration of WBC displayed a rebound reaction reaching its maximum at day 15 in all four strains at both aging stages (Table 3).

**Table 3:** Rebound maxima of blood count parameters after 5-Fluorouracil treatment

Maxima		mtAKR		B6Ntac		mt129S1		mtNOD	
		d	fold	d	fold	d	fold	d	fold
WBC	3m	15	1.3 ± 0.2	15	**1.8 ± 0.3**	15	1.2 ± 0.3	15	1.3 ± 0.5
	12m	15	1.8 ± 0.3	15	1.6 ± 0.4	15	1.5 ± 0.5	15	1.8 ± 1.1
Neutro	3m	18	1.4 ± 0.4	18	2.2 ± 4.0	15	1.8 ± 2.6	15	1.7 ± 1.4
	12m	15	2.7 ± 1.7	15	3.8 ± 3.1	15	2.7 ± 1.8	15	4.4 ± 2.9
Lymph	3m	15	1.3 ± 0.3	15	**1.8 ± 0.3**	15	1.3 ± 0.3	15	1.3 ± 0.5
	12m	15	1.7 ± 0.2	15	1.5 ± 0.4	15	1.5 ± 0.5	15	1.7 ± 1.1
Mono	3m	15	3.3 ± 4.0	18	2.3 ± 2.3	18	3.7 ± 3.3	15	1.5 ± 0.8
	12m	15	5.0 ± 4.5	15	5.5 ± 4.5	21	2.3 ± 1.4	18	3.9 ± 4.3
RBC	3m	21	0.9 ± 0.2	18	1.0 ± 0.1	21	0.9 ± 0.1	21	1.0 ± 0.1
	12m	18	1.0 ± 0.1	18	**0.8 ± 0.1**	18	0.9 ± 0.1	18	0.9 ± 0.1

Values of blood parameters are given as ratio to corresponding control group with standard deviation. (d) indicates the first day of maximal restoration after reaching the nadir. Bold values indicate a significant difference to mtAKR strain at same aging stage and day after 5-FU administration. Each group consisted of 10 animals. Of these, half was sacrificed at day 15 and the other half at day 21 for further investigations of BM.

Rebound reactions of total WBC as well as within the lymphocyte compartment were significantly stronger in young B6Ntac mice compared to the mtAKR strain. RBC recovery was similar in all strains at both aging stages with nadir between days 9 and 12 and restoration without rebound reaction nearly to the initial value. However, B6Nac mice exhibited a lower nadir at three months at day 9 and slower regeneration at 12 months at days 15 and 18 compared to mtAKR.

Moreover, 12 month-old mt129S1 mice displayed a higher nadir of neutrophils at day 6 and a faster regeneration at day 12 than mtAKR strain (Figure 4B). Additionally, the mt129S1 strain displayed a slower regeneration of monocytes at 3 and 12 months than mtAKR animals with significantly lower regeneration at day 15 and a delayed rebound maximum at day 21.

The pancytopenic and regeneration phases in mtNOD were quite similar to the mtAKR strain (data not shown). Notably, two mtNOD mice died unexpectedly within the experiment between days 9 and 10. WBC of these animals was extremely low in the prior blood count (0.32 and 0.44 x10^3^/μl).

The investigation of BM cells at day 15 and 21 did not display considerable differences between the four strains concerning ROS and ATP levels as well as the subpopulations in the BM (data not shown).

## DISCUSSION

**Figure 5:**
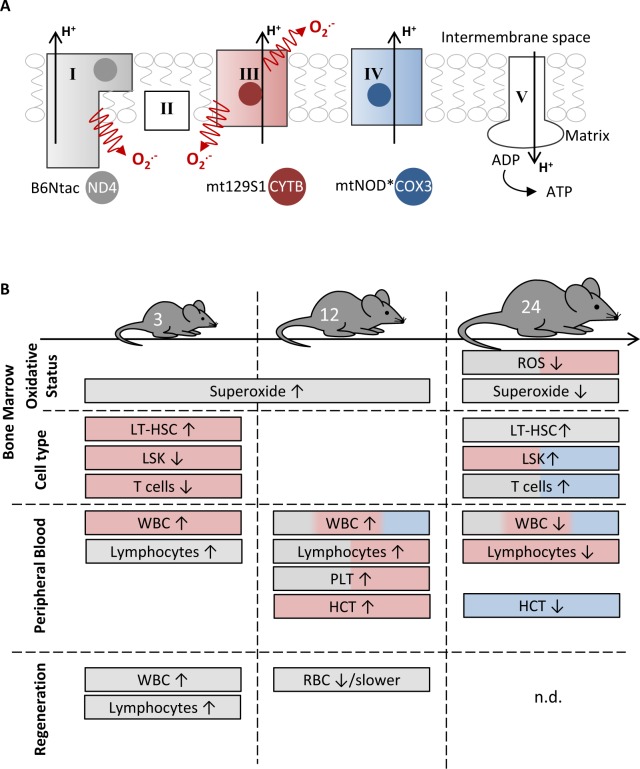
The impact of distinct mtDNA polymorphisms on hematopoiesis at different aging stages **A.** The respiratory chain consists of five complexes. Of these, three (complexes I, III and IV) participate in the generation of the proton gradient across the inner membrane. The mtAKR mouse strain served as reference, because the analyzed strains B6Ntac (grey), mt129S1 (red) and mtNOD (blue) differ in a single polymorphism affecting one subunit of complex I, III and IV, respectively. *The mtNOD strain harbors an additional polymorphism in tRNA-Arg. **B.** The figure summarizes the main features of each strain in comparison to the reference mtAKR.

The aging of the hematopoietic system is characterized by changes in blood compositions and cell function [17]. Furthermore, aging is accompanied by an accumulation of mtDNA mutations correlating with a possible mitochondrial dysfunction [8,9]. The impact of distinct mtDNA variations on hematopoietic aging can be investigated by using conplastic mouse strains. Here, we characterized three mouse strains (B6Ntac, mt129S1, mtNOD) harboring specific polymorphisms in the mtDNA influencing complex I, III and IV of respiratory chain in regards to their changes in hematopoiesis during aging. The impact of these polymorphisms on BM and peripheral blood is summarized in Figure 5.

First, ROS and superoxide levels during aging were analyzed. Our data reveal that intracellular ROS levels in B6Ntac and mtNOD significantly decline during aging mostly due to a decline of mitochondrial superoxide production.

The *ND4* protein, which is altered in the B6Ntac strain, is a hydrophobic inner membrane subunit of respiratory chain complex I. It is proposed to participate in the proton translocation function of this complex [18,19]. Distinct alterations in the human *ND4* gene were linked to known mitochondrial diseases like MELAS (mitochondrial myopathy, encephalopathy, lactic acidosis, and stroke-like episodes) and Leber's hereditary optic neuropathy, which are associated with very short lifespan [20]. Animals in our study did not show such severe phenotypes. Nevertheless, the polymorphism in *ND4* gene did impact the hematopoietic system.

Additionally, there was a significant higher proportion of CD34^dim/−^ cells in LSK pool of 24 month-old mice in B6Ntac compared to mtAKR. The absence of the antigen CD34 or its diminished expression, respectively, are characteristics of LT-HSCs (long-term-HSCs) in mice [21]. Ito et al. reported that elevated ROS levels drive HSCs out of quiescence and reduce maintenance capacities of the HSC pool [22]. Furthermore, it was shown that murine LT-HSCs containing low levels of ROS remain quiescent with higher maintenance capacity and less cycling compared to LT-HSCs with higher ROS content [23].

Based on this data, one could assume that peripheral blood counts during aging would be higher as well as recovery following cytostatic stress would be faster compared to mtAKR, respectively. However, this was not the case. Further studies will therefore have to resolve the molecular changes caused by the ND4 gene polymorphism in the B6Ntac mice in order to elucidate the biological effects.

*CYTB* is the only subunit of complex III encoded in mtDNA. Mutations in *CYTB* might be followed by a failing assembly of complex III leading also to negatively impacted complex I function [24,25]. In our study, the conplastic mouse strain mt129S1, harboring polymorphism nt15124 A/G, displayed significantly reduced ROS levels compared to mtAKR at 24 months. ROS levels within the mt129S1 strain did not change during aging. This data is in line with recent results from Mayer et al. and Schauer et al. who investigated the strain in regards to learning and fibroblast aging [26,27]. Despite a percentage wise increase of LSK cells in the BM this did not result in an increased WBC nor an improved recovery following cytotoxic stress compared to mtAKR. In fact, as in B6Ntac a significant decrease of peripheral blood lymphocytes was observed during aging. Therefore an impact of *CYTB* polymorphism on lymphopoiesis seems present. To our knowledge, this association has not been described earlier and needs further explorations.

The third strain we analyzed comparatively to mtAKR was the mtNOD strain, harboring a polymorphism in *COX3*, which is part of the catalytic core of complex IV [28]. *COX3* was also shown to be essential for complex assembly [29]. Our data demonstrate a significant and continuous decrease of intracellular ROS production in mtNOD BM cells during aging. Mutation in COX3 mtDNA have been described to cause encephalopathy, myopathy, lactic acidemia and exercise intolerance [30] as well as cramps and myoglobinuria [31]. Data on effects in hematopoiesis are limited.

Knock down of subunit Vb of complex IV with significantly lower complex activity in murine macrophage cell line showed that complex IV deficiency led to elevated ROS levels as well as reduced ATP levels [32].

During aging the hematopoiesis is characterized by specific changes in blood count, such as a decreasing WBC [33] and a shift towards myeloid cells [34–36]. The detected lower WBC due to a decreased lymphocyte fraction is in line with these findings. However, in case of the mtNOD strain, a possible impact of the additional polymorphism in tRNA-Arg should be taken into account.

We further investigated hematopoietic restoration capacity after 5-FU administration. Interestingly, young mice of the B6Ntac strain revealed a significantly stronger rebound reaction concerning WBC and lymphocytes in restoration phase after 5-FU administration in comparison to mtAKR mice. Numazawa et al. [37] revealed enhanced oxidative stress in BM cells after 5-FU treatment in mice. Keeping the glutathione pool constant, on the other hand, could diminish hemotoxicity [37]. Further, a stronger proliferation potential in mammalian cells following elevated ROS levels has already been demonstrated in different cell types [38–40]. However, an enhancement of proliferation and differentiation, respectively, in HSC was shown to lead to exhaustion of the stem cell pool [22, 23].

In conclusion, all three polymorphisms in mtDNA had some impact on hematopoiesis as summarized in Figure 5. However, observed changes in BM cell ROS production did not lead to significant uniform shifts of peripheral blood counts or altered responses to cytostatic stress. The fact that no consistent influence was found might point to an indirect, possibly hormetic action of the varying ROS levels. Further investigations are needed to explore the distinct impact of the polymorphisms in the whole respiratory chain complexes. In particular, the influence on lymphopoiesis should be in focus as well as the mechanism by which ROS levels are reduced in aged cells.

## MATERIALS AND METHODS

### Animals

Conplastic mouse strains were implemented as described before [16]. In short, due to the maternal inheritance of mtDNA, female animals of the mitochondrial donor strains AKR/J, 129S1/SvlmJ and NOD/LtJ, respectively, were crossed with males with the requested genomic background (C57BL/6NTac). Following female offspring was bred with males of the background strain. After ten generations the conplastic mouse strain harbors a stable mtDNA of the donor strain as well as a stable genomic DNA of the background strain. Animals were bred in the central animal facility of the University of Rostock. For *in vitro* analysis of BM and blood count animals with age of 3, 12 and 24 months were investigated. For *in vivo* study animals at 3 and 12 months were examined. Animal experiments were approved by Animal Care Committee of Mecklenburg-Western Pomerania (LALLF M-V/TSD/7221.3-1.1-100/12).

### Isolation of BM cells

Animals were sacrificed under narcosis (Ketamine 65 mg/kg BW and Xylazine 13 mg/kg BW) by cervical dislocation. Tissue was removed from femora and tibiae, which were cut at both sides and flushed several times with PBS. Cells were collected in falcon tubes and kept on ice until further procedure.

### Blood count

After retrobulbar collection whole blood was analyzed by the Advia^®^ system (Siemens Healthcare, Erlangen, Germany). Blood samples were standardly diluted by factor 4 in saline directly before measurement.

### Measurement of ROS and ATP levels

For measurement of complete intracellular ROS BM cells were incubated with 50 μM 2′,7′-dichlorofluorescine diacetat (DCFH-DA, Sigma, Taufkirchen, Germany) for 30 min. Fluorescence of 5×10^4^ cells was measured at Glomax^®^ (Promega, Mannheim, Germany) at a wavelength of 485 nm.

BM cells were stained with MitoSOX^TM^ (MS; Invitrogen, Darmstadt, Germany) to evaluate the amount of cells containing mitochondrial superoxide. After staining with 10 nM of MS for 10 min at 37°C, cells were incubated in 100 μl Annexin binding buffer with 4 μl Annexin-V-APC (Beckton Dickinson, Heidelberg, Germany). Cells were analyzed at the FACSCalibur (Beckton Dickinson, Heidelberg, Germany) and the proportion of non-apoptotic, MS positive cells was counted.

Total intracellular ATP levels were analyzed by using the ATP Bioluminescence Assay Kit HS II (Roche, Mannheim, Germany). In brief, luminescence of cell lysates of 5×10^4^ cells mixed with automatic injection of luciferase reagent was measured in triplicates at Glomax^®^ (Promega, Mannheim, Germany). By using a standard serial dilution of ATP the concentration in cell lysates could be determined.

### Immunophenotyping

To characterize the proportions of the different BM subpopulations the cells were stained with fluorescent antibodies (all obtained from Beckton Dickinson, Heidelberg, Germany) against different surface antigens in distinct panels: lymphocytes (IgG2 anti-CD3ε PE (Cat.-No. 553240), IgG2a anti-CD45R FITC (Cat.-No. 553087)); erythroid cells (IgG2b anti-Ter-119 APC (Cat.-No. 557909), IgG1 anti-CD71 PE (Cat.-No. 553267)); stem cells (IgG2b anti-c-kit PE (Cat.-No. 553355), IgG2a anti-Sca-1 PE-Cy7 (Cat.-No. 558162), lineage cocktail APC (Cat.-No. 558074), IgG2a anti-CD34 FITC (Cat.-No. 560238)) and corresponding isotype controls (hamster-IgG2 PE (Cat.-No. 550085), iso lineage cocktail APC (Cat.-No. 558074), rat-IgG2a FITC (Cat.-No. 553929), rat-IgG2a PE-Cy7 (Cat.-No. 557855), rat-IgG2b PE (Cat.-No. 556925), rat-IgG1 PE (Cat.-No. 554685)). Stained cells were measured with FACSCalibur (Beckton Dickinson, Heidelberg, Germany). Total nucleated cells (TNC) were gated and the amount of cells positive for the distinct antigens was evaluated.

### *In vivo* studies

Young (3 month old) and mid-aged (12 month old) mice were treated with 5-Fluorouracile (5-FU; i.p. 150 mg/kg BW) to evaluate the self-renewable capacity of HSCs after cytostatic stress. A control group treated with the same volume of isotonic saline was carried along for every strain and aging stage. Study groups consisted of 10 mice. For blood count whole blood (50 μl) was taken every 2 to 4 days by tail bleeding up to day 15 and 21, respectively. After 15 or 21 days animals were sacrificed and BM was isolated and investigated as described above. Furthermore, spleen weight was measured. Data of the 5-FU treated groups were normalized to the corresponding control.

### Statistical analysis

Results within each experiment were described using mean and standard deviation. Significance between strains was calculated using Mann-Whitney U test. A p-value <0.05 was considered to be significant.

## SUPPLEMENTARY MATERIALS FIGURES AND TABLES


