# Supplementary material for: Polymorphisms of the murine mitochondrial ND4, CYTB and COX3 genes impact hematopoiesis during aging
Source: Oncotarget. 2016 Sep 10;7(46):74460–72. doi: 10.18632/oncotarget.11952 (PMC5342679; doi:10.18632/oncotarget.11952)
Supplement: Supplementary file 1 [file oncotarget-07-74460-s001.pdf]

# Polymorphisms of the murine mitochondrial *ND4*, *CYTB* and *COX3* genes impact hematopoiesis during aging

## Supplementary Material

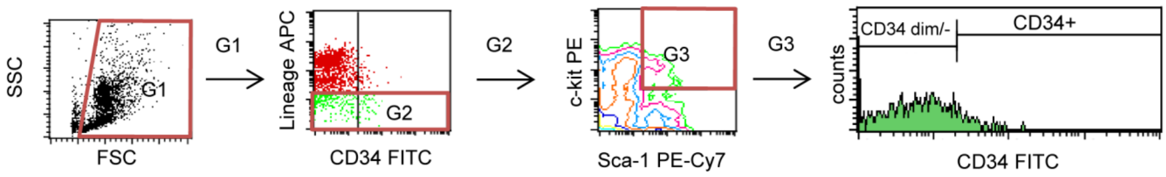


**Supplement 1:** **Analysis of LSK cells.**

**Supplement 2: Numbers of animals.**

**Table 1: Numbers of animals for ROS, Mitosox and ATP level analysis.**

| Parameter | Strain | 3 month | 12 month | 24 month |
|---|---|---|---|---|
| ROS | mtAKR | 6 | 6 | 7 |
| | B6/Ntac | 10 | 6 | 8 |
| | mt129S1 | 6 | 9 | 15 |
| | mtNOD | 9 | 6 | 13 |
| Mitosox | mtAKR | 6 | 7 | 8 |
| | B6/Ntac | 6 | 6 | 11 |
| | mt129S1 | 8 | 6 | 8 |
| | mtNOD | 7 | 6 | 6 |
| ATP | mtAKR | 7 | 6 | 8 |
| | B6/Ntac | 9 | 6 | 6 |
| | mt129S1 | 6 | 6 | 13 |
| | mtNOD | 9 | 6 | 16 |

**Table 2: Numbers of analyzed animals for immunophenotyping.**

| Parameter | Strain | 3 month | 12 month | 24 month |
|---|---|---|---|---|
| LSK cells CD34dim/- [%of LSK] CD3+ CD45+ CD11b | mtAKR | 6 | 6 | 8 |
| | B6/Ntac | 7 | 6 | 9 |
| | mt129S1 | 7 | 6 | 7 |
| | mtNOD | 6 | 7 | 10 |

**Table 3: Numbers of animals for blood count analysis.**

| Parameter | Strain | 3 month | 12 month | 24 month |
|---|---|---|---|---|
| Blood count | mtAKR | 7 | 22 | 15 |
| | B6/Ntac | 22 | 21 | 16 |
| | mt129S1 | 19 | 19 | 30 |
| | mtNOD | 22 | 21 | 28 |
